# Age-related variations in left ventricular diastolic parameters assessed automatically from phase-contrast cardiovascular magnetic resonance data: comparison against doppler echocardiography

**DOI:** 10.1186/1532-429X-14-S1-P155

**Published:** 2012-02-01

**Authors:** Alban Redheuil, Emilie Bollache, Golmehr Ashrafpoor, Eric Bruguière, Arshid Azarine, Ludivine Perdrix, Julien Dreyfus, Benoit Diebold, Elie Mousseaux, Nadjia Kachenoura

**Affiliations:** 1 Cardiovascular Imaging, HEGP European Hospital Georges Pompidou - Université Paris Descartes, Paris, France; 2 Functional Imaging Laboratory LIF, INSERM U678, UPMC, Paris, France; 3 Cardiology, HEGP European Hospital Georges Pompidou - Université Paris Descartes, Paris, France

## Summary

Relationship with age and comparison of phase-contrast-CMR and Doppler-echocardiography derived left ventricular diastolic function parameters in asymptomatic individuals with preserved ejection fraction.

## Background

Recent studies suggest the ability of phase-contrast cardiovascular magnetic resonance (PC-CMR) to provide velocity and flow-related left ventricular diastolic function parameters in good agreement with Doppler echocardiography (DE). Furthermore, DE diastolic parameters are known to change with aging. The aim of the present study is to assess whether the age-related variations of diastolic parameters are comparable when assessed by DE and PC-CMR.

## Methods

We studied 80 asymptomatic volunteers (50 males, mean age 44.7+/-16.7 years [19-79]) who underwent DE and PC-CMR exams on the same day. Transmitral EDE and ADE and lateral mitral annulus E’DE peak velocities were assessed by DE.

For PC-CMR analysis, a custom software was used for semi-automated segmentation of mitral annulus velocities and transmitral flow throughout the cardiac cycle and for automated extraction of diastolic parameters from velocity and flow rate curves. Flow rate curves provided: 1) early diastolic peak filling rate (EfMR, ml/s) and peak atrial filling rate (AfMR, ml/s), 2) peak filling rate to filling volume ratio (EfMR/FVfMR, s-1), and 3) deceleration time (DTMR), while maximal velocity curves provided the early to late peak velocities ratio EMR/AMR. Myocardial velocity curves provided peak early diastolic E’MR myocardial longitudinal velocity.

## Results

Table 1 summarizes mean values of diastolic parameters obtained by DE and PC-CMR as well as their respective correlations with age. Highly significant relationships were obtained either when using DE (EDE/ADE; EDE/E’DE) or PC-CMR (EMR/AMR ; EfMR/AfMR; EfMR/FVfMR; DTMR; EMR/E’MR). Slightly stronger correlations were obtained in PC-CMR parameters such as: EfMR/AfMR (r = 0.67); EfMR/FVfMR (r=0.66) and EMR/E’MR (r = 0.62) and in DE for E’ (r=0.71 ), all p<0.0001.

**Table 1:** Relationship with age of diastolic velocity and flow parameters. Comparison of PC-CMR and Doppler-Echocardiography.

	Mean±SD	Correlation with age	p value
**Echocardiographic measurements**			

E_DE_/A_DE_	1.23±0.50	0.64	<0.0001
E_DE_/E'_DE_	5.57±1.75	0.47	<0.0001
E'_DE_ (cm.s^-1^)	14.2±4.4	0.71	<0.0001
DT_DE_ (ms)	181±48	0.21	0.07

**CMR measurements**			

E_MR_/A_MR_	1.26±0.43	0.57	<0.0001
Ef_MR_/Af_MR_	1.29±0.58	0.67	<0.0001
Ef_MR_/FV_MR_ (s^-1^)	3.93±0.90	0.66	<0.0001
E_MR_/E'_MR_	7.89±4.69	0.62	<0.0001
E'_MR_ (cm.s^-1^)	8.9±4.2	0.68	<0.0001
DT_MR_ (ms)	201±52	0.55	<0.0001

## Conclusions

Our automated method provided PC-CMR diastolic parameters which were strongly related to age. These age-related variations of diastolic parameters appear to be comparable when assessed by DE or PC-CMR with a slight superiority of the PC-CMR flow-related parameters. These findings suggest the usefulness of PC-CMR diastolic data when analyzed automatically as an additional CMR tool for the evaluation of left ventricular function.

## Funding

Nothing to disclose.

